# Diagnostic work-up strategy for periprosthetic joint infections after total hip and knee arthroplasty: a 12-year experience on 320 consecutive cases

**DOI:** 10.1186/s13037-015-0071-8

**Published:** 2015-05-16

**Authors:** Dirk Zajonz, Lena Wuthe, Solveig Tiepolt, Philipp Brandmeier, Torsten Prietzel, Georg Freiherr von Salis-Soglio, Andreas Roth, Christoph Josten, Christoph-E. Heyde, Mohamed Ghanem

**Affiliations:** Department of Orthopaedic Surgery, Traumatology and Plastic Surgery, University Hospital Leipzig, Liebigstrasse 20, 04103 Leipzig, Germany; Department of Nuclear medicine, University Hospital Leipzig, Liebigstrasse 20, 04103 Leipzig, Germany; Department of Diagnostic and Interventional Radiology, University Hospital Leipzig, Liebigstrasse 20, 04103 Leipzig, Germany

**Keywords:** Prosthetic joint infection, Diagnosis, THA, TKA

## Abstract

**Background:**

Elective knee and hip arthroplasty is followed by infections in currently about 0.5–2.0 % of cases – a figure which is on the increase due to the rise in primary implants. Correct diagnosis early on is essential so that appropriate therapy can be administered. This work presents a retrospective analysis of the diagnoses of patients suffering infections after total hip or knee arthroplasty.

**Methods:**

320 patients with prosthetic joint infection (PJI) following knee or hip arthroplasty were identified. They comprised a) 172 patients with an infection after total hip arthroplasty (THA): 56 % females (n = 96) and 44 % males (n = 76) with a mean age of 70.9 (39–92) years; and 148 patients with an infection after total knee arthroplasty (TKA): 55 % females (n = 82) and 45 % males (n = 66) with a mean age of 70.7 (15–87) years.

**Results:**

Although significantly more TKA than THA patients reported pain, erythema, a burning sensation and swelling, no differences between the two groups were observed with respect to dysfunction, fever or fatigue. However, significant differences were noted in the diagnosis of loosening (THA 55 %, TKA 31 %, p < 0.001) and suspected infection using conventional X-rays (THA 61 %, TKA 29 %, p < 0.001). FDG-PET-CT produced very good results in nearly 95 % of cases. There were no differences between THA and TKA patients regarding levels of inflammation markers. Histological evaluation proved to be significantly better than microbiological analysis.

**Summary:**

The clinical picture may be non-specific and not show typical inflammatory symptoms for a long time, particularly in PJI of the hip. As imaging only provides reliable conclusions after the symptoms have persisted for a long time, morphological imaging is not suitable for the detection of early infections. FDG-PT-CT proved to be the most successful technique and is likely to be used more frequently in future. Nevertheless, there are currently no laboratory parameters which are suitable for the reliable primary diagnosis of PJI. Diagnosis requires arthrocentesis, and the fluid obtained should always be examined both microbiologically and histologically.

## Introduction

Hip and knee replacements are among the most common operations worldwide. The quantity performed is increasing significantly in response to demographic change. For example, the number of total hip arthroplasties (THA) carried out in the US rose by a factor of 2.5 from 200,216 in 1993 to 497,419 in 2005. In the same period, the amount of primary total knee arthroplasties (TKA) grew 1.7-fold from 135,992 to 237,645 [1,2]. High expectations are attached to arthroplasty, which has been described as the operation of the century [3] due to its high success rate. Although the risk of complications (as with any medical procedure) cannot be excluded, the implantation of endoprostheses leads to problems in fewer than 10 % of cases [4]. The main drawback occurring is aseptic loosening [4]. Although infections occur more rarely, they are one of the most feared complications and are potentially fatal. Reflecting the quantity of primary joint replacements, the number of infections diagnosed in the US has steadily increased: between 1990 and 2004, the number of hip arthroplasty infections more than tripled while knee arthroplasty infections increased almost six-fold [5].

Thanks to the introduction of perioperative antibiotic prophylaxis and the optimization of operating hygiene (such as the use of cleanroom standards in the operating theatre), infection rates following elective knee and hip arthroplasty have been reduced from about 10 % in the 1960s to currently about 0.5–2.0 % [4,6-8]. However, this rate climbs to about 5 % after revision surgery, and to as high as 15–40 % after reimplantations [4,9,10]. Infection creates a high psychological, physiological and financial burden for the patient, their family members, the attending physicians and cost carriers. Even if treatment is successful in a single operation, the antibiotic additives alone may cost as much as €5000 [11]. For complex revision surgery, Barrack et al. reported treatment costs exceeding $50,000 back in 1999 [12].

Appropriate therapy depends on early, correct diagnosis. Given the usually enormous consequences often entailing explantation, diagnosis has to be as reliable as possible [13]. Since the clinical presentation of PJI varies greatly, a detailed medical history should be compiled addressing any existing risk factors [14]. In particular, the general clinical symptoms of inflammation may be non-specific in acute systemic or subacute local inflammation [14]. At any rate, pain in the replaced joint accompanied by fever needs to be diagnosed [9].

The aim of this study was to analyse retrospectively the diagnosis of patients with PJI in an attempt to identify any valid diagnostic parameters. Another goal was to identify any differences in the individual diagnostic factors concerning knee and hip arthroplasty infections.

## Material and methods

Patients were considered to suffer infection if clinical and paraclinical examination methods resulted in a positive microbiological culture or positive histological findings in arthrocentesis or surgery specimens indicating prosthetic joint infection (PJI).

To select the patient cohort, all patients with the ICD-10 diagnosis code T84.5 (infection and inflammatory reaction due to internal joint prosthesis) who had been treated in our department between 1 January 2001 and 31 December 2012 were retrospectively identified. Patient data were gathered from archived medical records and electronic records in IS-H (SAP) (Siemens AG Healthcare Sector, Erlangen, Germany) as well as from radiological findings and images in SIENET MagicWeb/ACOM (Siemens AG Healthcare Sector, Erlangen, Germany). From this patient population, a total of 320 patients with PJI after TKA or THA were identified in whom an infection of knee or hip arthroplasty was first diagnosed in the above-mentioned period in our department.

All in all, 172 patients with periprosthetic infection after THA were identified. Comprising 56 % (96) females and 44 % (76) males, their mean age when infection was diagnosed was about 70.9 (39–92) years. Early infections (within the first six weeks after THA) accounted for 32 % (55 patients), delayed infections (between six weeks and two years after THA) accounted for 27 % (47) of infections, while in 38 % (66), infection occurred over two years after THA. In four patients, the time of THA was unknown [7].

There were 148 cases of periprosthetic infection following TKA. The patient cohort consisted of 55 % (82) females and 45 % (66) males with a mean age of 70.7 (15–87) years. Early infection occurred in 20 % (29 patients), delayed infection affected 42 % (62), and in 36 % (54) infection only occurred after at least two years. For three patients, the exact period since surgery was unknown [7].

General clinical symptoms like pain, fatigue and fever as well as clinical findings such as erythema, burning sensation, swelling, dysfunction and fistula formation were evaluated. Furthermore, the presence of sepsis was ascertained. The inflammation parameters CRP (mg/l), leukocytes (Gpt/l), Hb (mmol/l) and sometimes PCT (ng/ml) were measured on admission or first presentation. Radiologists’ findings using conventional X-rays documenting described loosening or the suspicion of infection were taken into account. Suspected infections following analysis of CT, MRI, three-phase bone scintigraphy, anti-granulocyte scintigraphy or PET-CT (FDG-PET) by specialists were also included. Furthermore, the findings of microbiological and histological analyses of diagnostic arthrocentesis were compared to the definitive results during surgery.

Statistical analysis was performed using the spreadsheet software Microsoft Excel (Microsoft Corporation, Redmond, USA). The *t*-test for two dependent samples was used to calculate significance, the level of significance being set at 5 % (α = 0.05).

## Results

### Clinical diagnosis

**Fig. 1:**
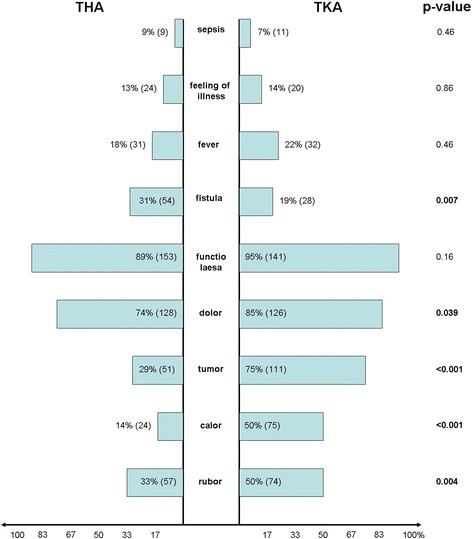
Clinical symptoms. Graph showing percentage and absolute difference in clinical symptoms between THA (left) and TKA (right) infection as well as the p-value (statistical significances printed in bold type)

The individual clinical symptoms are shown in Fig. 1. The high percentage of patients with THA or TKA infection suffering pain (TKA 95 % (141), THA 89 % (153)) and dysfunction in the joint concerned (THA 74 % (128), TKA 85 % (126)) was striking. Significantly more patients with TKA treatment reported pain than THA patients (p = 0.039). Furthermore, significant differences regarding erythema, burning sensation and swelling were reported by a higher proportion of patients with TKA infection (Fig. 1). There were no differences in the predominance of fever, fatigue or sepsis. Fistula formation occurred significantly more often following THA than TKA infection (THA 31 % (54), TKA 19 % (28); p = 0.0067).

### Laboratory diagnosis

Data for CRP, leucocyte count and haemoglobin were available in 168 cases of THA infection. In 14 cases, the procalcitonin level had also been measured. Laboratory data was available for 145 cases of TKA infection, including in 13 cases the level of procalcitonin.

Mean CRP in patients with THA infection was about 98.2 mg/l (min/max 0.89–438 mg/l) and mg/l 117.5 (1.12–515 mg/l) in patients with TKA infection. The mean leukocyte count was 9.67 Gpt/l (3.4–30.8 Gpt/l) in patients with THA infection and 10.57 Gpt/l (0.6–44 Gpt/l) in patients with TKA infection. Mean PCT was 2.94 ng/ml (0.07–15.16 ng/ml) in patients with THA and 11.08 ng/ml (0.03–100.3 ng/ml) in patients with TKA infection. Mean Hb was 6.93 mmol/l (3.2–9.2 mmol/l) in patients with TKA infection and 7.17 mmol/l (4.6–10.7 mmol/l) in patients with THA infection. The differences between patients with THA and TKA infections were not significant.

The individual laboratory readings are compared between THA and TKA infections in Table 1, which also lists the numbers of patients with abnormal laboratory data.

**Table 1:** Laboratory readings. Shows the laboratory readings compared between THA and TKA infections and also the numbers of patients with abnormal laboratory data

	Percentage of positive patients (absolutely)	Arithmetic average (min-max)	Standard	Arithmetic average (min-max)	Percentage of positive patients (absolutely)
CRP	95 % (159)	98.2 (0.89–438)	<5 mg/l	117.5 (1.12- 515)	93 % (135)
Leukocytes	39 % (65)	9.67 (3.4–30.8)	4–9 Gpt/l	10.57 (0.6- 44)	43 % (63)
Procalcitonin (PCT)	57 % (8)	2.94 (0.07–15.16)	<0.5 ng/ml	11.08 (0.03–100.3)	54 % (7)
Hemoglobin level	79 % (133)	6.93 (3.2–9.2)	8.1 – 10.7 mmol/l	7.17 (4.6–10.7)	74 % (108)

### Imaging diagnostics

**Fig. 2:**
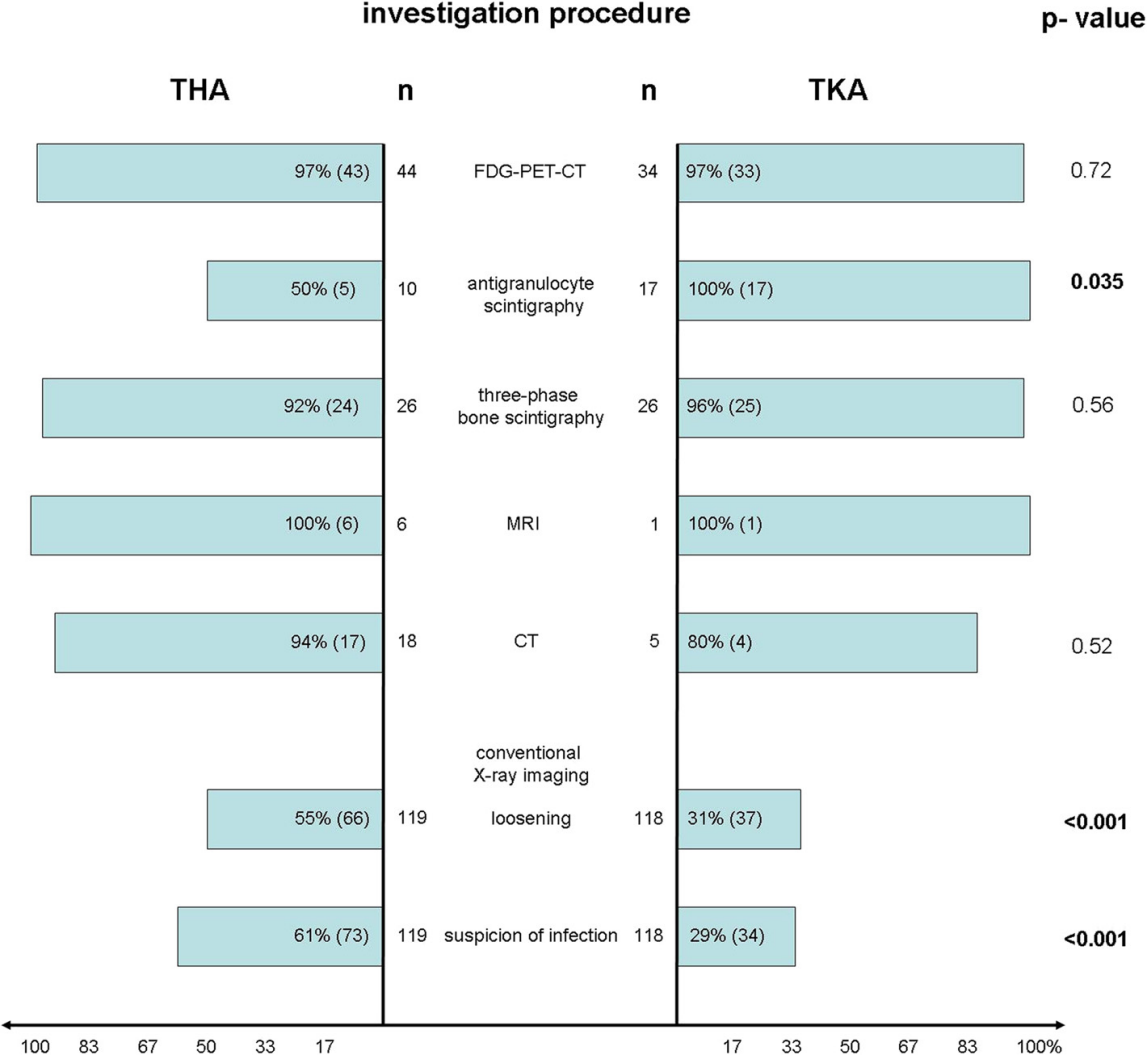
Imaging techniques. Graph showing percentage and absolute difference of imaging techniques comparing their success rates at detecting THA (left) and TKA (right) infections as well as the p-value (statistical significances printed in bold type)

A detailed list of imaging techniques comparing their success rates at detecting THA and TKA infections is contained in Fig. 2.

**Fig. 3:**
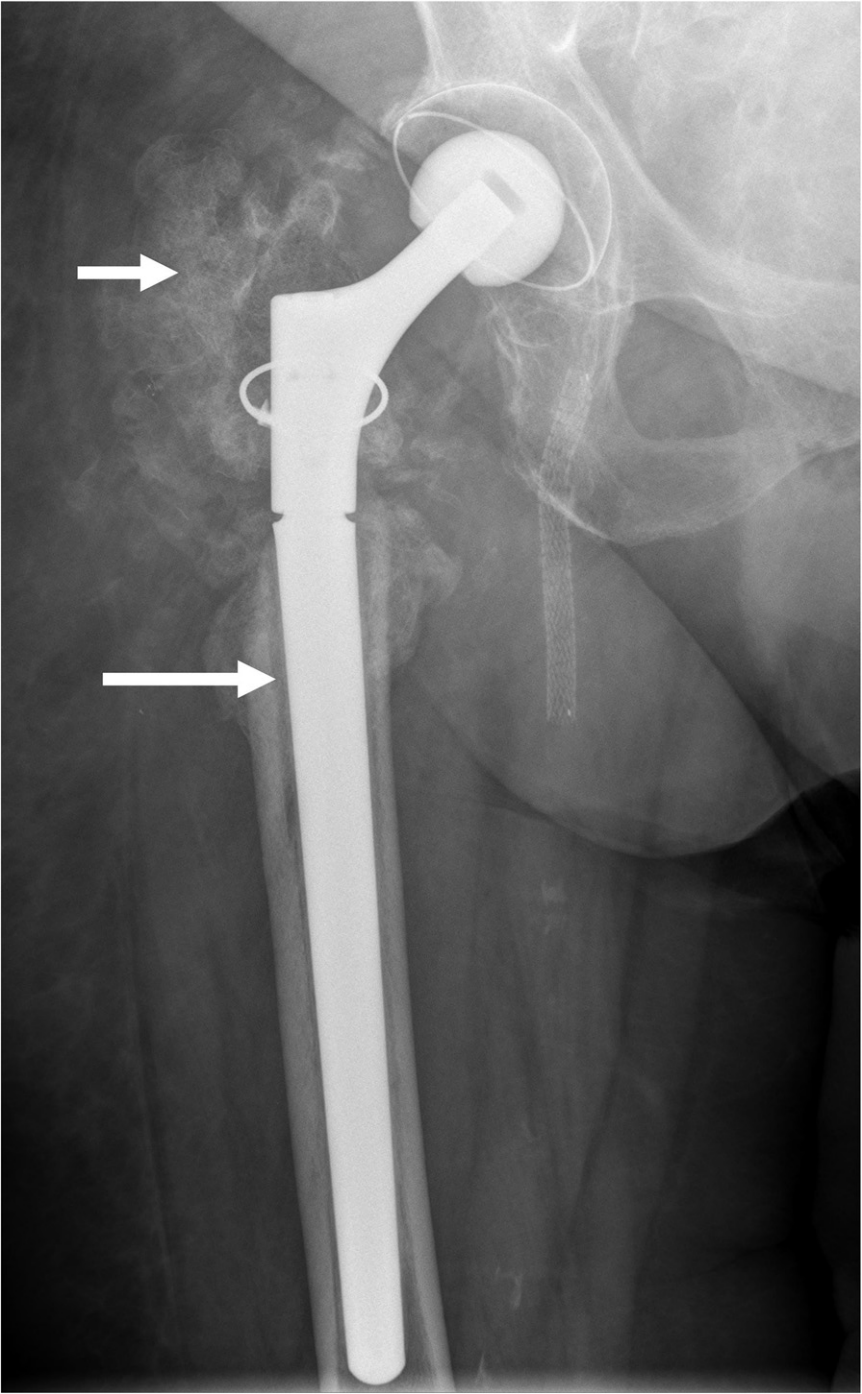
Conventional hip X-rays. Showing a conventional hip X-rays with existing infection with loosening of the implant (long bolt) and periarticular ossifications (short bolt)

In a total of 119 conventional hip X-rays (n = 73), infection was suspected in 61 % (n = 73) while loosening of the implant was revealed in 55 % (n = 66). Knee X-rays were taken on 118 patients. Suspected infection was found in 29 % of cases (n = 34). Signs of loosening were apparent in 31 % (n = 37) Fig. 3.

Significant differences emerged between THA and TKA in the detection of implant loosening (THA 55 % (66), TKA 31 % (37); p <0.001) and suspicion of infection by a radiologist (61 % (73), TKA 29 % (34); p <0.001), with infection after THA more likely to be detected.

CT scans were produced of 18 hip joints, of which 94 % (17) showed signs of infection. CT scans were performed on 5 knee joints, with positive results being achieved in 80 % (4). There were no significant differences between the two groups in terms of CT (p = 0.52).

MRI was used for diagnosis even less frequently: in 6 cases on the hip joint and 1 case on a knee joint. All the MRI scans positively indicated suspected infection.

**Fig. 4:**
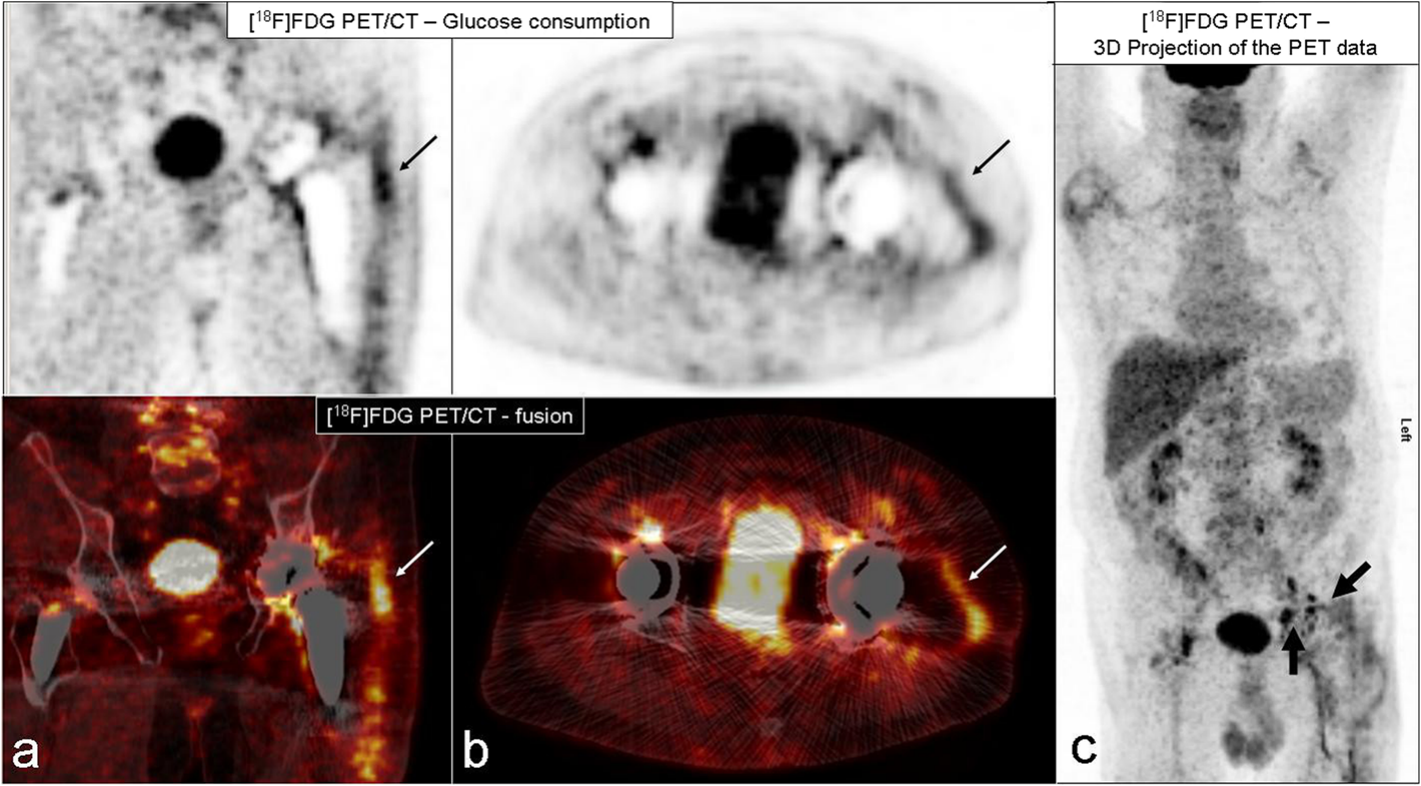
PET/CT images. Representative [18 F] FDG PET/CT images of a patient with infection of the left total hip arthroplasty in coronal **(a)** and transversal **(b)** orientation **(c)** shows a 3D projection (maximum intensity projection) of the [18 F] FDG PET data. The images demonstrate a streaky intensive hypermetabolism in the soft parts surrounding the total hip arthroplasty (thin arrows) and focal hypermetabolisms in reactive inguinal lymph nodes (bold arrows)

The most common type of nuclear medicine examination carried out was FDG-PET-CT. Of the 44 hip joints examined, infection was suspected in 97 % (43). Infection was also indicated by 33 of the 34 knee examinations (97 %). There were no significant differences (p = 0.72) Fig. 4.

**Fig. 5:**
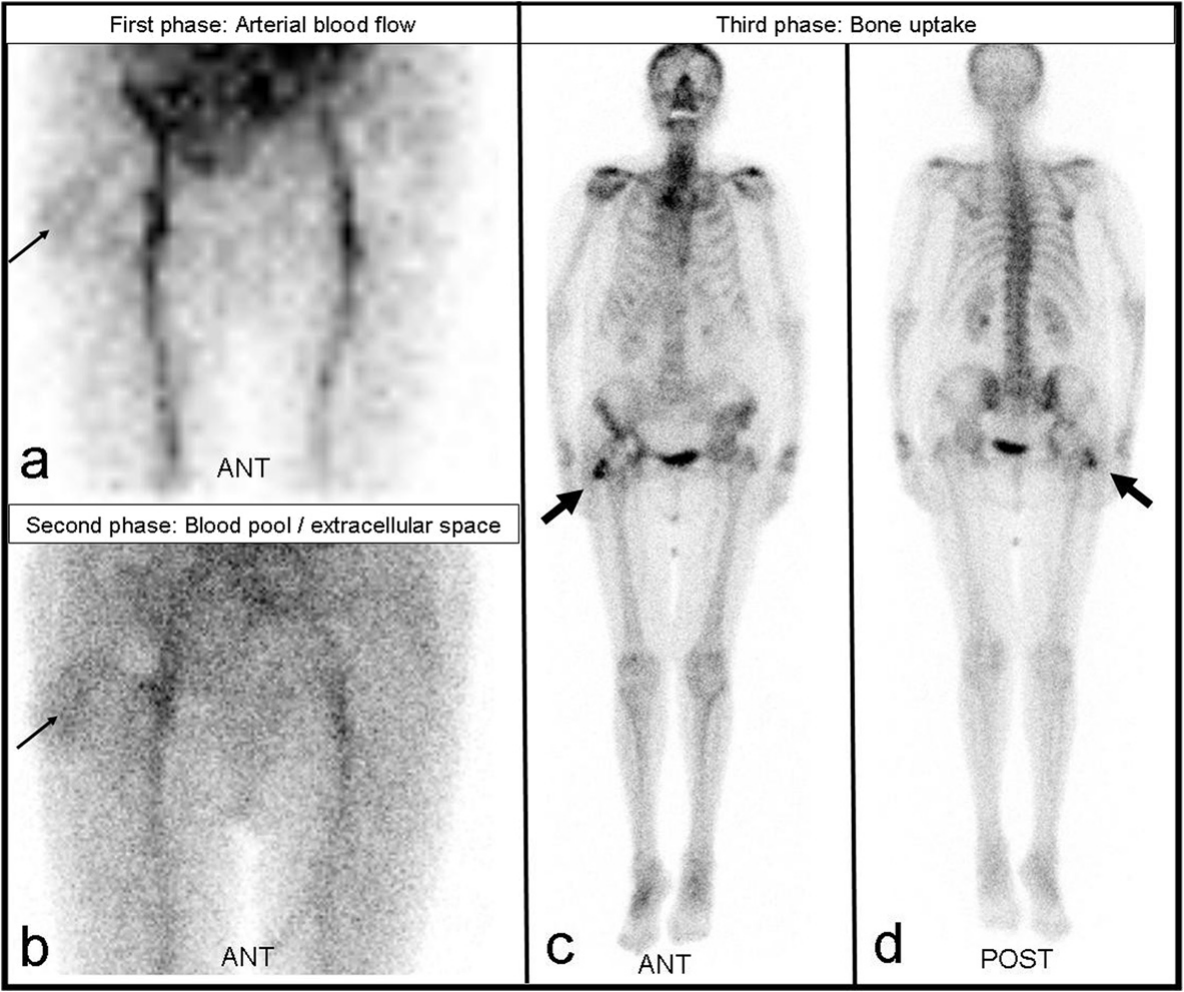
Three-phase bone scintigraphy. [99mTc] DPD three-phase bone scintigraphy. Increased blood flow **(a)** and blood pool **(b)** in the soft tissue of the right hip, lateral of the total hip arthroplasty (thin arrows) as well as increased bone uptake **(c, d)** in the lateral parts of the right hip (bold arrows) in a patient with infection of the right total hip arthroplasty

Three-phase bone scintigraphy was performed on 26 hips and 26 knees. It was positive in 92 % (n = 24) of hips and 96 % (n = 25) of knees (p = 0.56) Fig. 5.

Anti-granulocyte scintigraphy was carried out on 10 THA and 17 TKA patients. Significant differences were ascertained between THA and TKA regarding suspected infection according to a nuclear medicine specialist (THA 50 % (5), TKA 100 % (17); p = 0.035) in favour of TKA.

### Diagnostic arthrocentesis

Arthrocentesis was performed on 85 patients with THA infection. The specimens were all analysed microbiologically. In addition, 53 specimens underwent histological evaluation.

Arthrocentesis was performed on 115 patients with TKA infection. The specimens were all analysed microbiologically while histological valuation was also carried out on 74 specimens. The positive and negative findings for THA and TKA infections are compared in Table 2.

**Table 2:** Microbiology and histology. Shows the positive and negative findings (percentage and absolute) for THA and TKA infections in microbiology and histology as well as the p-value

	Microbiology		p-value		Histology	
	Positive	Negative			Positive	Negative
THA (n = 85)	63 (74.5 %)	22 (25.5 %)	0.019	THA (n = 53)	47 (88.7 %)	6 (11.3 %)
TKA (n = 115)	86 (74.8 %)	29 (25.2 %)	0.025	TKA (n = 74)	63 (85.1 %)	11 (14.9 %)

Regarding the correlation between microbiology and histology, of the 63 positive microbiological cultures following THA infection, positive histology was observed in 60 % (38 cases), too. However, in three cases (5 %), the histological findings were negative despite positive microbiological cultures. In 9 arthrocentesis specimens with no positive microbiological culture (41 %), histological findings still indicated suspected infection.

Following TKA infection, positive histology also occurred in 58 % (50 cases) of all positive microbiological cultures. By contrast, no histological signs of infection were found in 9 % (8) of cases. Of the negative microbiological cultures, 45 % (13 cases) nevertheless showed positive histology.

## Discussion

Strikingly, our study found that significantly more patients with TKA infection suffered swelling, erythema and a burning sensation in the affected joint than THA patients Fig. 1, whereas, hardly any differences existed concerning pain or dysfunction. This is because the smaller layer of soft tissue on the knee joint leads to symptoms of localized inflammatory response showing earlier [7] while non-specific symptoms such as pain and dysfunction are experienced on the hip before any local inflammatory reaction becomes visible on the skin [15]. However, no differences emerged between systemic signs of infection such as general malaise, fever or sepsis, the systemic involvement of which is possible in connection with both THA and TKA infection. On the other hand, fistulas were found to occur significantly more often in connection with THA infection. This is probably because the infection is often silent and only noticed when the fistula opens. In the knee joint, a local inflammatory response is much more likely, prompting the patient to seek medical advice and treatment before a fistula can develop [16]. Furthermore, the clinical symptoms of PJI vary greatly, affecting the general clinical symptoms of inflammation [14]. Especially in chronic cases, they may be non-specific and are often misinterpreted in connection with previous pain in the joint [14]. In fact, up to 30 % of prosthetic infections show no clinical symptoms whatsoever. Therefore, pain in the replaced joint accompanied by fever must always be properly diagnosed [9].

The first diagnostic tool used is mostly conventional X-ray. Although X-rays can detect loosened implants, they can only sometimes distinguish between septic or aseptic loosening, making them ineffective for early infections [17]. Moreover, any existing periarticular ossifications only show up after a long time and do not prove an existing infection [18]. In our analysis, X-rays revealed significantly more loosenings and suspected infections on replacement hips than knees – partly because symptoms following THA take longer to develop [14,16] Fig. 3.

Although magnetic resonance imaging (MRI) is considered the technique of choice for soft tissue assessment in inflammatory events, interference from adjacent metallic implants impedes accurate assessment [19]. Substantial interference has even been reported in connection with non-ferromagnetic implants, such as titanium [20]. Therefore, MRI only seems suitable for specific problems such as abscesses in soft tissue [21]. CT also shows significant metal artefacts and therefore only plays a minor role in the diagnosis of PJI. It may be useful to assess any resulting changes to bone or to plan a possible explantation, but its use should be decided on a case-by-case basis [14,22].

Nuclear medicine techniques are still held in high regard and produce good or even very good results regarding specificity and sensitivity in the diagnosis of PJI, despite the varying nature of data in the literature [23]. The problem is that they are usually only useful at least six months postoperatively, making them unsuitable for diagnosing early infection [24].

Three-phase bone scintigraphy is a common and relatively inexpensive technique. It detects the enrichment of ^99m^technetium-labelled diphosphonates on the bone surface at three different times by means of a gamma camera. This enrichment depends on bone remodelling and is therefore more intensive if the endoprosthesis becomes loose. Accordingly, although this method is sensitive, it is also rather unspecific and provides no diagnostic evidence of a septic event, especially in the first twelve months after implantation [24,25]. Our study attributed showed good results to bone scintigraphy, the proportion of positively detected findings in THA and TKA exceeding 90 %. Then again, its accuracy is reported in the literature to be only 50–70 % [24,25] Fig 5.

In anti-granulocyte scintigraphy, ^99m^Tc-labelled monoclonal anti-granulocyte antibodies are used, which detect the presence of granulocytes in inflamed tissue. This method is reported in the literature to have very high sensitivity and specificity of 95–100 % [26]. This could not be confirmed in our study, especially regarding THA infections, as positive results were not revealed in 50 % of cases. The reason could not even be ascertained in a subsequent examination of the cases. There may be an anomaly involved which can partly be explained by the low number of cases of anti-granulocyte scintigraphy assessed (n = 10). By the way, two disadvantages of anti-granulocyte scintigraphy are that it is expensive while its use of mouse antibodies sometimes triggers allergic reactions.

To improve specificity, scintigraphy can be combined with single-photon emission computed tomography (SPECT), a method which detects the distribution of a radioisotope in the body using planar imaging [26].

FDG-PET-CT showed especially good results in our study with 97 % correctly positive results in both THA and TKA. In the literature, however, the accuracy of FDG-PET-CT is reported to vary greatly between 43 and 92 % [27,28]. Furthermore, its high costs and time-consuming operation as well as the low proliferation of FDG-PET-CT scanners rule out its standard use in the diagnosis of PJI [29] Fig. 4.

Our study noted increased CRP levels in over 90 % of cases of both THA and TKA infections. The mean value in both types of infections was about 100 mg/l (<5 mg/l). Since CRP, being an acute phase protein, regularly increases non-specifically not just during infections but also after surgery, determining the CRP level is particularly suitable for monitoring development [30]. However, studies have found CRP to have a sensitivity of 91–93 % but a sensitivity of just 83–86 % in connection with infections [31].

Following arthroplasty, the increasing CRP reaches a maximum two or three days after surgery and returns to its normal range within three weeks [32]. A persistent rise or another increase are hence a sign of PJI and should always be properly diagnosed [19]. Further evidence of infection may be an increased white blood cell count (standard 4–10×10^6^/l), but this has low specificity [33]. However, fewer than half of all the patients in this survey showed an increase in leukocytes.

Procalcitonin and IL-6 are relatively new laboratory parameters with good specificity and sensitivity. But because only studies involving small numbers of cases have been performed so far, routine use is not recommended [34]. The advantage of procalcitonin is that it is a reliable marker of sepsis which allows the bacterial cause of sepsis to be diagnosed [34]. In our study, too, only about half the patients showed an increase in PCT. Although none of these laboratory parameters can reliably indicate or rule out PJI, they are important in order to monitor development [30]. Strikingly, over 70 % of patients displayed falling Hb values, which can probably be attributed to infectious anaemia. This, too, is extremely nonspecific, but appeared to be more sensitive in our study than leukocytes and PCT.

Ultimately, only positive microbiological culture is regarded as a decisive criterion for diagnosis. The gold standard here is arthrocentesis [10]. Even so, the sensitivity and specificity of the results can vary greatly and depend on the type and quantity of extracted fluid and ongoing antibiotic therapy. Moreover, valid conclusions are often only possible after fourteen days’ incubation, limiting its usage in acute diagnosis. The significantly higher positive values of histology compared to microbiology were noticeable regardless of whether the knee or the hip was involved (Table 2). This may be due to variations caused by antibiotic therapy beforehand or the insufficient amount of fluid extracted for microbiological cultivation [30]. Ultimately, our survey showed histological analysis to be clearly superior to microbiological evaluation. The main advantage of microbiological analysis is that it can quickly detect the pathogen present and its corresponding antimicrobial resistance so that drug-based therapy can start [30].

### Limitations

The validity of this study is reduced by its retrospective design and the inhomogeneity of the patient population. Then again, this applies to the majority of patients with PJI. There was no exact differentiation of diagnosis depending on the time of infection, which may be particularly significant for imaging.

## Conclusion

In particular, the clinical picture of PJI may be non-specific and, especially on the hip, not be accompanied by typical symptoms of inflammation. In imaging, reliable diagnosis is often only possible after some time, making the morphological representation of early infections difficult. FDG-PT-CT proved especially useful and is likely to become even more important. There are currently no laboratory parameters which are suitable for the reliable primary diagnosis of PJI. However, the traditional inflammatory markers, especially CRP, are essential to monitor the course of infection. Arthrocentesis is obligatory to diagnose infection, and specimens should undergo both microbiological and histological examination.

### Consent

All patients were informed and confirmed their approval.
